# Tetrahydrocurcumin Protects Microglial Cells Against *Pseudomonas aeruginosa* Lipopolysaccharide-Induced Reactive Oxygen Species Production and Cathepsin B to Activate NLRP3 Inflammasome-Mediated Pyroptosis Via the HO-1 and p38/JNK Pathway

**DOI:** 10.7150/ijms.118252

**Published:** 2025-09-21

**Authors:** Hui-Wen Lin, Tzu-Chun Chen, Inga Wang, Jui-Hsuan Yeh, Shang-Chun Tsou, Lee Sun, Shih-Chi Chung, Chen-Ju Chuang, Yuan-Yen Chang

**Affiliations:** 1 Institute of Medicine, Chung Shan Medical University, Taichung 40201, Taiwan.; 2 Department of Medical Research, Chung Shan Medical University Hospital, 40201, Taichung, Taiwan.; 3 Rehabilitation Sciences & Technology, University of Wisconsin-Milwaukee, Milwaukee, WI, 53211, USA.; 4 Department of Microbiology and Immunology, School of Medicine, Chung-Shan Medical University, and Clinical Laboratory, Chung Shan Medical University Hospital, Taichung 40201, Taiwan.; 5 Department of Optometry, Asia University, Taichung 413, Taiwan.; 6 Department of Nursing, Chung Shan Medical University, Taichung, 40201, Taiwan.; 7 Emergency department, St. Martin De Porres Hospital, Chiayi, 60069, Taiwan.

**Keywords:** Tetrahydrocurcumin (THC), Pseudomonas aeruginosa LPS (P.a. LPS), NLRP3, inflammasome, pyroptosis, BV-2 cells

## Abstract

Tetrahydrocurcumin (THC), a major curcuminoid metabolite known for its antioxidant and anti-inflammatory properties, has potential for reducing brain inflammation. This study investigated THC's anti-inflammatory responses and molecular mechanisms against* Pseudomonas aeruginosa* LPS (P.a. LPS)-induced NLRP3 inflammasome and ROS-producing cell pyroptosis. In the current study, we showed that THC significantly attenuated P.a. LPS-induced inflammasome factor IL-18 production through lysosomal dysfunction and leakage of cathepsin B. Importantly, THC reduced the levels of NLRP3 inflammasome and pyroptosis-related proteins, including NLRP3, active caspase-1, ASC, N-GSDMD, and IL-18. We also demonstrate that the inhibition of NLRP3 inflammasome activation by THC is ROS-dependent, via inhibition of H_2_O_2_ production, SOD activity, and enhancement of GSH activity. Subsequently, we demonstrated that THC treatment significantly reduced LPS-induced phosphorylation of c-Jun N-terminal kinase (JNK), p38 mitogen-activated protein kinase (MAPK), and increased heme oxygenase-1 (HO-1) expression in BV-2 cells. Furthermore, we used MAPKs (SB203580, SP600125, and U0126) and HO-1 (SnPP) inhibitors to demonstrate that THC modulated inflammasome-mediated pyroptosis may be related to p38 and JNK MAPK and HO-1-dependent inflammatory signaling. Overall, THC can inhibit ROS-triggered NLRP3 inflammasome-mediated pyroptosis by promoting GSH activity and HO-1 expression via modulating the p38 and JNK signaling pathways in P.a. LPS-treated BV-2 cells.

## 1. Introduction

The prevalence of neurodegenerative diseases, particularly Parkinson's and Alzheimer's disease, has markedly increased in recent decades 1, 2. While the precise mechanism remains elusive, excessive microglial activation has emerged as a contributing factor to the pathological progression of these conditions and the cascade of inflammatory responses in the brain 3. Activated microglia can release molecules that activate the inflammasome leading to induced pyroptosis in nearby cells, thereby promoting the accumulation of amyloid beta (Aβ) and neurofibrillary tangles in the brain 4. Conversely, attenuating microglia activation has shown promise in restoring synaptic integrity and improving functional outcomes in neurodegenerative diseases. This underscores the pivotal role of microglial immune responses in the exacerbation of neuroinflammation and neurodegenerative diseases 5.

Oxidative stress is also induced during inflammation and can modulate microglial activity. In the context of exogenous encephalitis infection, characterized by the presence of bacterial lipopolysaccharide (LPS), a study conducted by Park and colleagues revealed that LPS is proficient in elevating the levels of mitochondrial reactive oxygen species (ROS) in microglia 6. This increase subsequently triggers the production of tumor necrosis factor-α (TNF-α), interleukin (IL-1β, IL-6), inducible nitric oxide synthase (iNOS), and cyclooxygenase-2 (COX-2) through mitogen‑activated protein kinase (MAPK signaling pathways extracellular signal-regulated kinase (ERK), c-Jun N-terminal kinase (JNK), and p38 MAPK) pathway regulation 6. In another study, Han et al. Using quercetin inhibits LPS-induced mitochondrial ROS-mediated NLRP3 inflammasome activation in microglia by promoting mitochondrial autophagy 7. Pyroptosis and oxidative stress can perpetuate each other; for example, pyroptosis can lead to the release of cellular contents, including reactive oxygen species, which can exacerbate oxidative stress 6. A notable mechanism through which this threat manifests is the activation of the NOD-like receptor protein 3 (NLRP3) inflammasome during the occurrence of pyroptosis 8. Pyroptosis is a programmed cell death pathway that is often associated with inflammation, characterized by the activation of proinflammatory factors, such as IL-1β, IL-18, and NLRP3, the formation of pores in the cell membrane, and the release of cytoplasmic contents, which can trigger an immune response. However, cytokine maturation and release are dependent on Gasdermin D (GSDMD) in pyroptotic cells 9. Conversely, oxidative stress can promote the activation of inflammasomes and the induction of pyroptosis, leading to a cascade of inflammatory events and potential harm to the central nervous system 4, 9, 10. Therefore, understanding these interactions is crucial for developing therapeutic strategies to mitigate the progression of those diseases.

Tetrahydrocurcumin (THC), the major active metabolite of the natural antioxidant curcumin, is highly bioavailable 11, 12 and bioactive and has antioxidant, anti-inflammatory 13, and neuroprotective effects 14. We previously used a nitric oxide assay to demonstrate the protective effects of THC against *Pseudomonas aeruginosa* LPS (P.a. LPS)-induced hyperactivation of microglia and discovered that THC inhibited the STAT/JAK signaling pathway-mediated activation of inflammatory factors 15. Studies have also found that THC exhibits protective potential against septic cardiomyopathy by reducing oxidative stress and inflammation through modulation of JNK/ERK signaling 16. Although previous studies have illustrated the benefits of THC in antioxidant and anti-inflammatory related diseases, its antioxidant and anti-inflammatory effects on LPS-induced microglia have not been fully studied. Therefore, we aimed to investigate whether the role of THC in P.a. LPS-induced reactive oxygen species production to activate NLRP3 inflammasome-mediated pyroptosis is regulated via the HO-1 and MAPK pathway in BV-2 cells (a mouse microglial cell line).

## 2. Materials and Methods

### 2.1. Chemicals

SB203580 (p38 inhibitor, Cat. S1076), U0126 (ERK-1/2 inhibitor, S1102), and SP600125 (JNK inhibitor, S1460) were purchased from Selleck Chemicals (Houston, TX, USA). Tin protoporphyrin IX (SnPP; HO-1 inhibitor, Cat. HY101194) was purchased from MedChemExpress (NJ, USA).

### 2.2. Microglial cell culture

The murine microglial BV-2 cell line was obtained from Dr. Dah-Yu Lu (China Medical University, Taichung, Taiwan), who purchased the cell line from the American Type Culture Collection (Manassas, VA, USA). The cells were cultured in RPMI 1640 medium (Gibco, NY, USA) containing 10% fetal bovine serum (No.10437, Gibco) and 100 U/mL penicillin-100 U/mL streptomycin (Anti-Anti; Gibco). All cells were cultured at 37°C in an atmosphere of 5% CO_2_ in air. BV-2 cells were seeded in 12-well plates overnight at 2.5 × 10^5^ cells/well before treatment to ensure attachment. Cells were pretreated with different concentrations of THC (0, 10, 20, and 40 μM) for 1.5 h and then treated with *P. aeruginosa* LPS (0.1 μg/mL) for subsequent analysis.

### 2.3. Cell viability assay

After the designed reaction, 10 μL of CCK-8 reagent (Dojindo Molecular Technologies, Japan) was added to each group of BV-2 cells, which were then dissolved in 500 μL of RPMI medium and incubated at 37°C for 30 min. Next, 200 μL of the supernatant was transferred to a 96-well plate, and absorbance at 450-595 nm was quantified using a Multiskan SkyHigh microplate spectrophotometer (Thermo Fisher Scientific, Waltham, MA, USA). All percentages were calculated as absorbance values compared with the control.

### 2.4. Enzyme-linked immunosorbent assay

After the designed reaction, the cultured medium of each group was centrifuged at 12,000 × g for 5 min. Extracellular IL-18 levels were measured by using a sandwich ELISA kit (DY122-05, Lot 340606; R&D Systems, MN, USA) according to the manufacturer's instructions.

### 2.5. Measurement of intracellular ROS levels

After the designed reaction, the cell culture medium of each group was replaced with 10% RPMI containing 2 μM 2',7'-dichlorodihydrofluorescein diacetate (H_2_DCF-DA; No. 15240; AAT Bioquest, Pleasanton, CA, USA) and incubated at 37°C for 30 min. BV-2 cells were separated by 0.25% trypsin-EDTA (No. 25200056, Thermo Fisher Scientific), and their absorbance at 485 nm was measured using a NovoCyte flow cytometer (Agilent, CA, USA). All percentages were calculated as absorbance values compared with the control.

### 2.6. Measurement of antioxidant enzyme activity and hydrogen peroxide levels

After the designed reaction, the cell lysates of each group were collected, and the activities of SOD (Cat. 706002), catalase (Cat. 707002), and glutathione (Cat. 703002) were measured using detection kits purchased from Cayman (Ann Arbor, Michigan, USA). The cultured medium was centrifuged at 12,000 × g for 5 min, and hydrogen peroxide (H_2_O_2_) levels were measured using an assay kit (K265200, Biovision Research, Milpitas, CA, USA). All percentages were calculated as values compared with the control.

### 2.7. Acridine orange staining

After the designed reaction, the cell culture medium of each group was replaced with 10% RPMI containing 5 μM acridine orange (ab270791, Abcam, UK) and incubated at 37°C for 30 min. BV-2 cells were separated using 0.25% trypsin-EDTA (No. 25200056, Thermo Fisher Scientific), and absorbance at 615 nm (red fluorescence) was measured using a NovoCyte flow cytometer (Agilent). All percentages were calculated as absorbance values compared with the control.

### 2.8. Annexin V and propidium iodide staining

After the designed reaction, BV-2 cells were separated using 0.25% trypsin-EDTA and treated with 0.25 µg/mL annexin V-fluorescein isothiocyanate and 1 µg/mL propidium iodide (PI) for 1 h in the dark. Absorbance was measured using a flow cytometer. Annexin V-fluorescein isothiocyanate was detected at 485 nm, and PI was detected at 615 nm.

### 2.9. Western blotting

After the designed reaction, cell lysates were diluted in RIPA buffer and boiled at 95°C for 5 min. Proteins from each group were separated using SDS-PAGE (8%-12%) and transferred to polyvinylidene fluoride membranes (Pall Life Sciences, NY, USA). The membranes were soaked in 5% nonfat milk (in 1x PBST) for 2 h and then incubated with primary antibodies GSDMD (Lot.5500013333, ABclonal, MA, USA), NLRP3 (NBP2-12446, Novus Biologicals, CO, USA), AIM2 (B8, Santa Cruz), ASC (F-9, Santa Cruz, Tx, USA), cleaved-caspase-1 (Asp 927, Santa Cruz), IL-1β (ab9722, Abcam), IL-18 (ab191152, Abcam), and GAPDH (6C5, Santa Cruz) overnight at 4 °C. The membranes were then washed and incubated with a secondary antibody (anti-rabbit/mouse IgG) conjugated with horseradish peroxidase (Thermo Fisher Scientific) for 1 h. Proteins were detected using an enhanced chemiluminescence primer (Cytiva, MA, USA). Protein intensities were quantified using AlphaImager 2200 (Alpha Innotech, San Leandro, CA, USA), and folds were compared with a control group.

### 2.10. Statistical analysis

The groups and enrollees in this study were randomly assigned. Calculations were performed in Microsoft Excel. Groups were compared using one-way analysis of variance (ANOVA) and post hoc tests of least significant difference. Statistical significance was indicated by p < 0.5.

## 3. Results

### 3.1. THC decreased NLRP3 inflammasome-mediated pyroptosis in the P.a. LPS-induced BV2 cells

**Figure 1:**
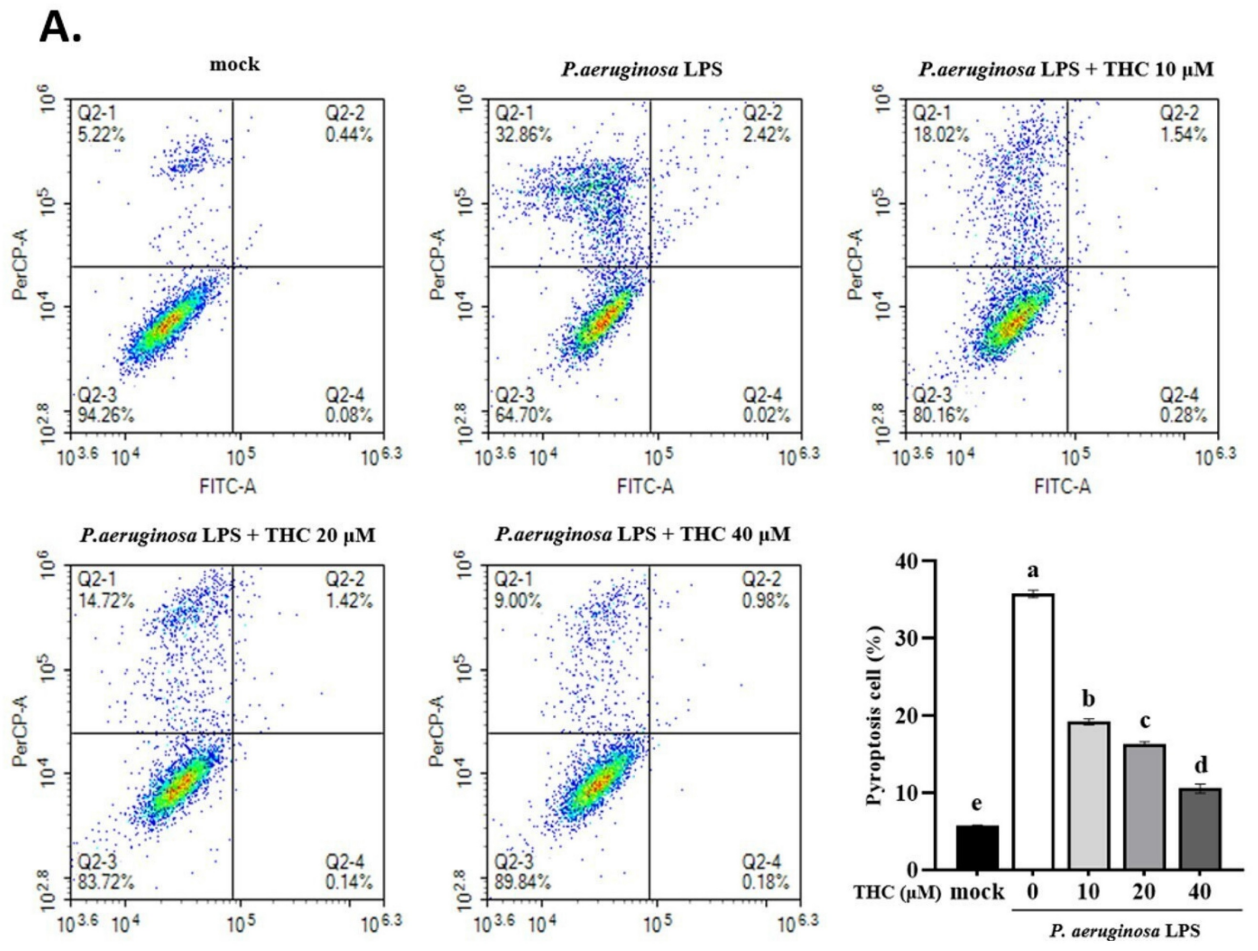

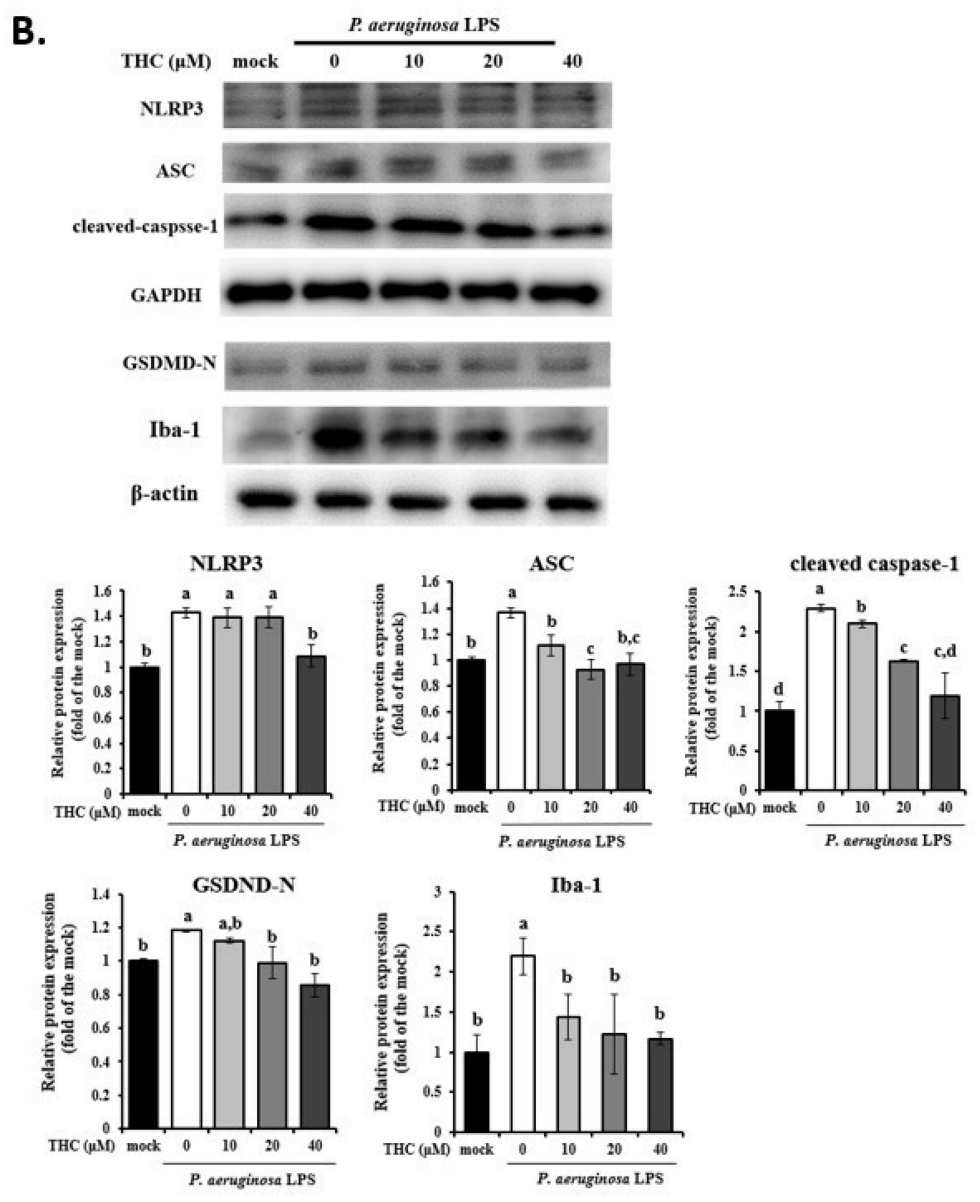
Effects of THC on pyroptosis production in P.a. LPS-treated BV-2 cells. The BV-2 cells were pretreated with THC (0, 10, 20, or 40 µM) for 1.5 h, and then treated with 0.1 µg/mL P.a. LPS for 24 h. (A.) The BV-2 cells were stained with annexin V and propidium iodide (PI) to isolate pyroptotic cells. The cells were divided into four regions {Q2-1 = annexin V (-), PI (+); Q2-2 = annexin (+), PI (+); Q2-3 = annexin V (-), PI (-); Q2-4 = annexin V (+), PI (-)}, and pyroptotic cells were counted (Q2-1). The pyroptotic cells were expressed as a percentage compared with the mock. (B.) The cell lysates were analyzed to evaluate proteins associated with NLRP3 inflammasome via western blotting. Relative expression of proteins was expressed as a fold compared with the control group (mock). All data are presented as mean ± standard deviation (n = 3). a-e with the same symbol means no statistical difference was observed between groups (p < 0.05).

THC is known to exert anti-inflammatory effects by inhibiting the expression of pro-inflammatory cytokines, but its mechanism of action is still unclear, whether it is through blocking NLRP3 inflammasome activation and cell pyroptosis 17. As demonstrated in Fig. 1A, the rate of pyroptosis (annexin V-/PI+) in the P.a. LPS-treated BV-2 cells was significantly higher than the mock group; however, cells pretreated with 40 µM THC were significantly lower than the P.a. LPS-treated group. We also collected BV-2 cell lysates to analyze protein expression by western blotting. We found that THC protected P.a. LPS-induced BV-2 cells notably downregulated the NLRP3, decreased cleavage-caspase-1, and ASC protein expression (Fig. 1B). Previous studies have shown that inflammatory caspase-induced gasdermin D (GSDMD) activation leads to increased expression of N-terminal GSDMD (GSDMD-N) protein, which induces cell pyroptosis 18. To further confirm whether THC can affect the pathogenesis of P.a. LPS-induced pyroptosis, we performed western blotting to study its GSDMD-N manifestations. The data showed that the P.a. LPS-induced BV-2 cells have higher GSDMD-N expression than the mock groups, but THC pretreatment led to a significant reduction of its expression. Moreover, THC was found to reduce the Iba-1 in P.a. LPS-induced BV-2 cells (Fig. 1B). These results suggest that THC can regulate the activation of BV-2 cells by inhibiting P.a. LPS-induced NLRP3 inflammasome leading to cell pyroptosis.

### 3.2. THC suppresses cathepsin B (CTSB) protein to activate inflammasomes-derived IL-18 secretion in P.a. LPS-induced BV-2 cells

**Figure 2:**
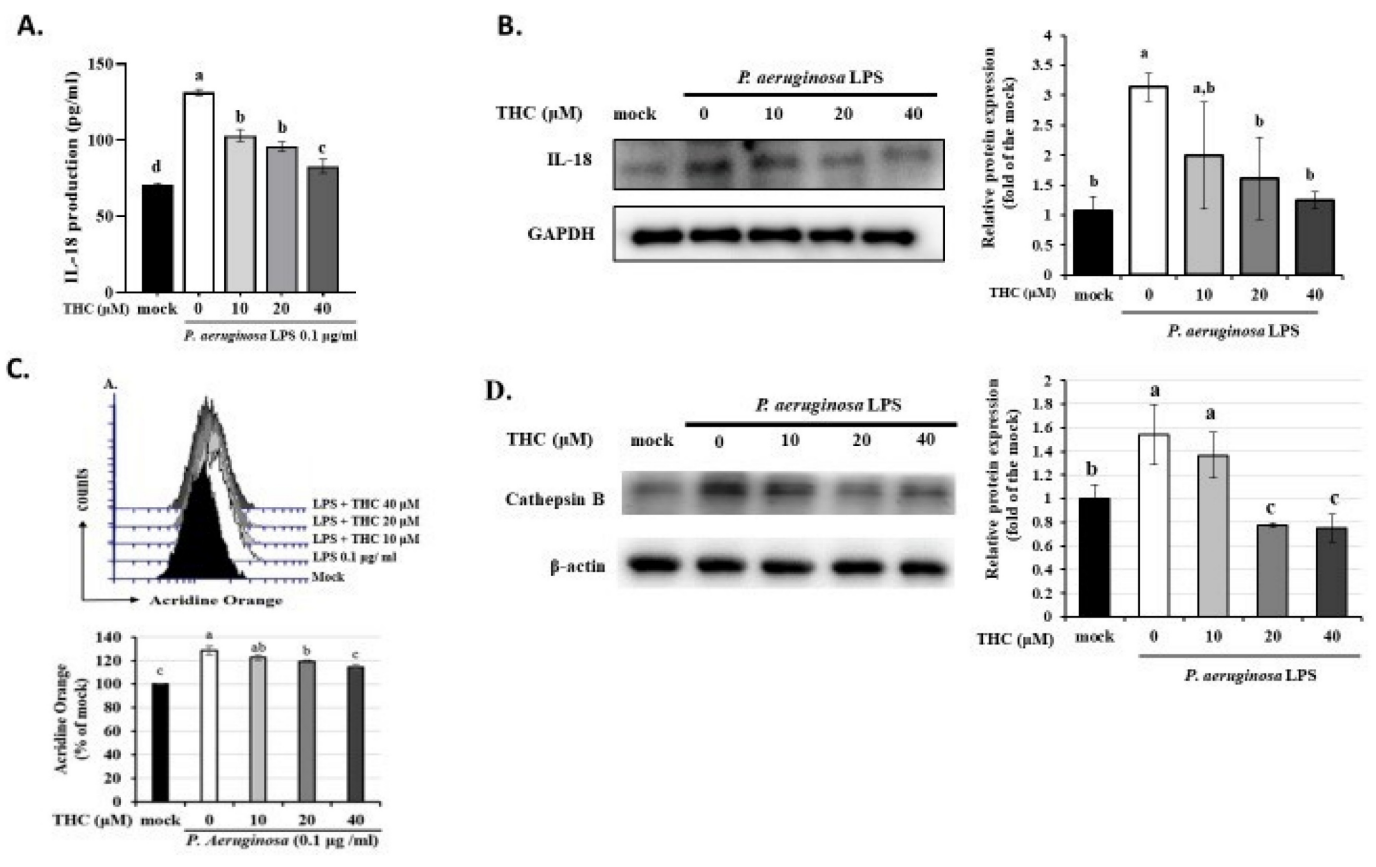
Effects of THC on the regulation of cathepsin B (CTSB) protein to activate inflammasomes-derived IL-18 secretion in P.a. LPS-treated BV-2 cells. The BV-2 cells were pretreated with THC (0, 10, 20, or 40 µM) for 1.5 h, and then treated with 0.1 µg/mL P.a. LPS for 24 h. (A.) ELISA was used to detect the production of IL-18 in the cell culture supernatants. (B.) The cell lysates were analyzed to evaluate IL-18 via western blotting. Fluorescence intensity was quantified as a percentage compared with the control group (mock). Relative expression of proteins was expressed as a fold compared with the control group (mock). (C.) Acridine Orange staining for inflammasomes study by flow cytometry. (D.) The cell lysates were analyzed to evaluate cathepsin B expression via western blotting. a-d with the same symbol means no statistical difference was observed between groups (p < 0.05).

We then used ELISA and western blotting to investigate whether THC can inhibit the formation of the inflammasome and thereby protect microglia by inhibiting the expression of IL-18 in P.a. LPS-treated BV-2 cells. According to Fig. 2A, P.a. LPS markedly increased extracellular IL-18 levels compared to the control group. However, as shown in Fig. 2B, pretreatment with THC significantly reduced IL-18 levels in a dose-dependent manner, with the most pronounced effect observed at the highest concentration (40 µM) of THC. These findings confirm that THC effectively suppresses microglial inflammatory responses.

Lysosomal dysfunction plays an essential role in NLRP3 inflammasome activation 19. Acridine Orange emits red fluorescence upon accumulation in intact lysosomes, but shifts to green fluorescence when released from ruptured lysosomes and dispersed into the cytosol and nuclei 20. To evaluate the effect of THC on the lysosomal membrane stability of P.a. LPS-treated BV-2 cells were analyzed by AO staining. As demonstrated in fig. 2C, P.a. LPS increased the acridine orange red fluorescence in BV2 cells; however, pretreatment with 40 µM THC decreased the fluorescence. Recent studies have shown that the integrity of the lysosomal membrane is disrupted, which may lead to the release of the lysosomal protease cathepsin B, thereby inducing NLRP3 activation 21. The results suggest that THC also significantly lowered the expression of cathepsin B in P.a. LPS-treated BV-2 cells (Fig. 2D). These findings indicate that THC might inhibit NLRP3 inflammasome activation by maintaining the integrity of lysosomes.

### 3.3. THC inhibited P.a. LPS-induced ROS production

**Figure 3:**
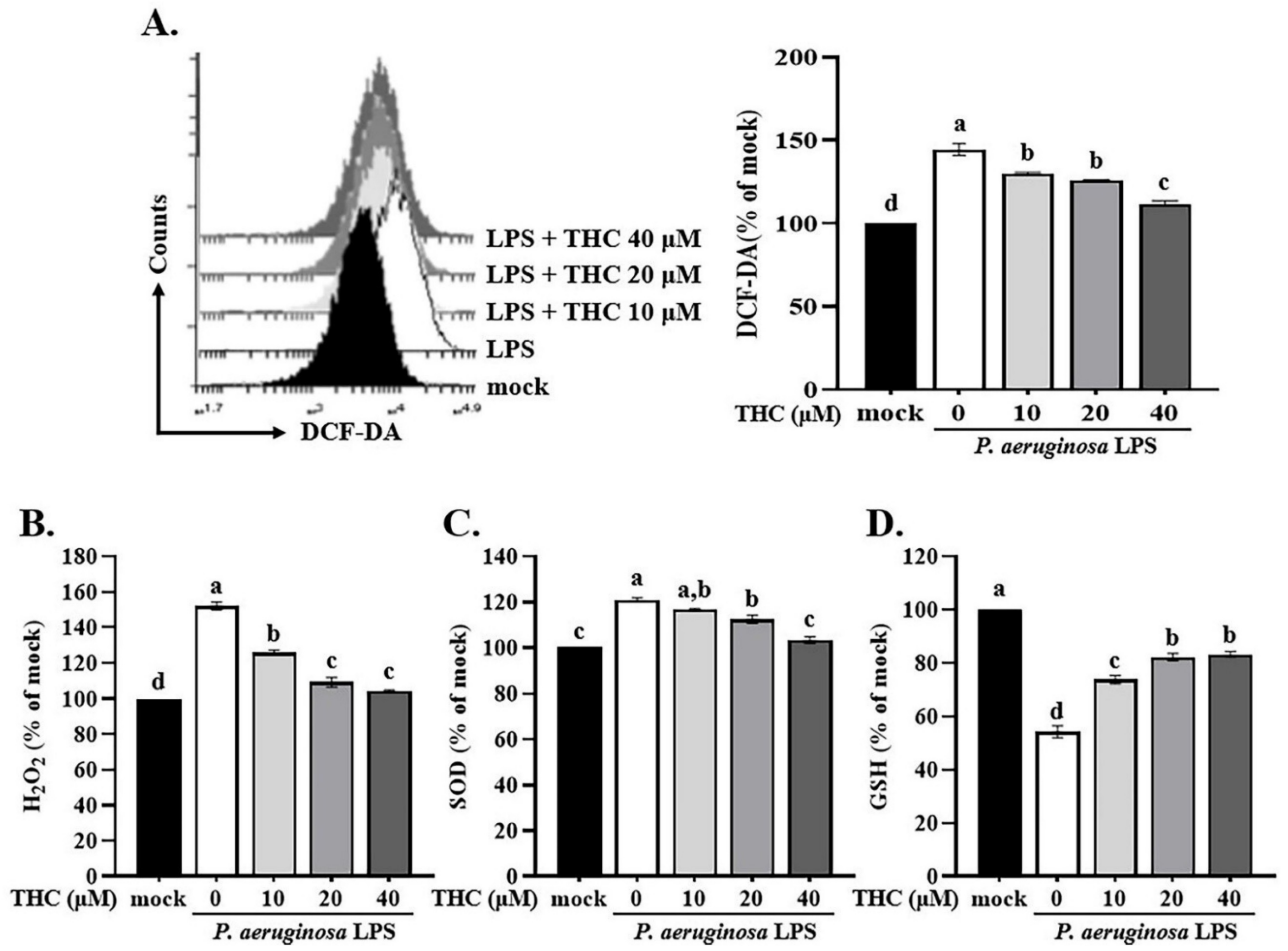
Effects of THC on ROS levels and antioxidant enzyme activity in P.a. LPS-treated BV-2 cells. After pretreatment with THC (0, 10, 20 or 40 µM) for 1.5 h and treatment with 0.1 µg/mL P.a. LPS for 24 h, **(A)** BV-2 cells in each group were stained with H_2_DCF-DA, intracellular ROS levels were examined through flow cytometry, and the activity levels of **(B)** H_2_O_2_,** (C)** SOD and** (D)** GSH were measured. a-d with the same symbol means no statistical difference was observed between groups (p < 0.05).

ROS are an important factor in immune regulation and a potential risk factor for an excessive inflammatory response 22. Previous studies have shown that NF-κB or ROS inhibitors attenuate the LPS-induced upregulation of NLRP3 mRNA, highlighting the critical involvement of NF-κB and ROS in the regulation of NLRP3 gene expression 23, 24. However, whether THC regulates ROS production or interferes with activation of the NLRP3 inflammasome is unclear. P.a. LPS increased ROS in BV-2 cells, as shown by stronger H2DCF-DA fluorescence. However, in cells pretreated with 40 μM THC, the fluorescence was reduced, indicating lower ROS levels (Fig. 3A).

H_2_O_2_ is a ROS that in high concentrations, can cause oxidative damage to cells. An imbalance between superoxide dismutase (SOD) and glutathione can lead to H_2_O_2_ levels increase and oxidative stress 25, which has been implicated in various diseases, including cancer, neurodegenerative disorders, and cardiovascular disease 24. To explore THC's regulation of P.a. LPS-induced ROS production in BV-2 cells, we examined the activity levels of antioxidant enzymes and intracellular H_2_O_2_ levels. BV-2 cells treated with P.a. LPS showed higher levels of H₂O₂ compared to the mock group (Fig. 3B). In addition, P.a. LPS increased the activity of the antioxidant enzyme SOD while decreasing GSH activity (Fig. 3C and D), which is consistent with the findings of Park and Chun, who reported that P.a. LPS contributes to microglial inflammatory response 26. However, pretreatment with 40 µM THC reversed these effects, reducing SOD activity and restoring GSH levels, suggesting a protective role of THC against oxidative stress in BV-2 cells.

### 3.4. THC reduced p38/JNK signaling pathway in P. a. LPS-induced BV-2 cells

The MAPKs/NFκB signaling pathway is a typical regulator of inflammatory responses. This pathway signals the activation of microglial inflammasomes under various stresses, according to various *in vivo* and *in vitro* models involving LPS-induced ischemia-reperfusion injury 19, 27. To assess whether THC regulated the activation of MAPK signaling pathways by P.a. LPS-induced BV2 cells, we used western blot and ELISA to examine the signal-related protein and inflammatory cytokines.

**Figure 4:**
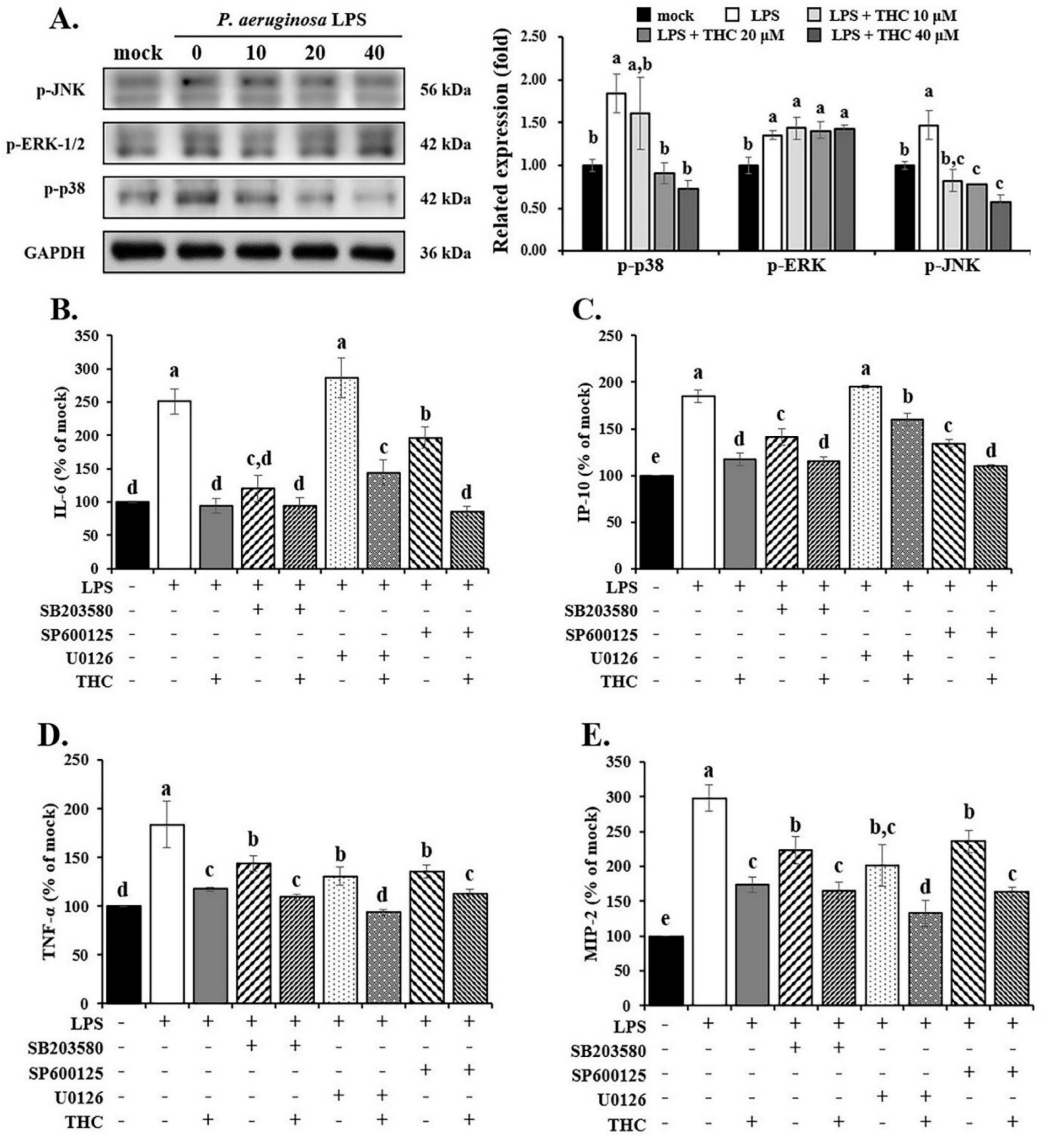
Effects of THC on P.a. LPS-mediated MAPK signaling pathways. (A) After pretreatment with THC (0-40 µM) for 1.5 h and treatment with 0.1 µg/mL P.a. LPS for 24 h, cell lysates in each group were collected to analyze the proteins expression of p-JNK, p-ERK and p-p38 through western blotting. After pretreatment with THC (0 or 40 µM), SB 203580 (0 or 10 µM), U0126 (0 or 10 µM), or SP 600125 (0 or 20 µM) for 1.5 h and treatment with 0.1 µg/mL P.a. LPS for 24 h, the levels of (B) IL-6, (C) IP-10, (D) TNF-α and (E) MIP-2 were measured using ELISA kits. The relative expression of proteins was expressed as a percentage compared with the control group (mock). a-e with the same symbol means no statistical difference was observed between groups (p < 0.05).

As shown in Fig. 4A, the protein expression of p-ERK-1/2, p-p38, and p-JNK was raised in P.a. LPS-treated BV-2 cells. However, in the THC experimental group, p-p38 and p-JNK expression were significantly reduced, especially in the 40 µM THC group, but no inhibition of p-ERK-1/2 was observed.

In addition, we also determined the effect of THC on the inflammatory factors by LPS-induced BV2 cells using ELISA. The results showed that using SB203580 (a p38 inhibitor, 10 µM) or SP600125 (a JNK inhibitor, 20 µM) 28, 29 on their own lowered the levels of IL-6, IP-10, TNF-α, and MIP-2 (Fig. 4B-E). In contrast, U0126 (an ERK inhibitor, 10 µM) 28 had little to no effect on IL-6 and IP-10 (Fig. 4B and C). However, when THC was added together with these inhibitors in LPS-treated BV-2 cells, it further reduced the levels of all four inflammatory markers more than the inhibitors alone.

### 3.5. THC prevented P. a. LPS-induced pyroptosis via the p38/JNK signaling pathway

To investigate whether THC affects the mechanisms of LPS-induced inflammation and pyroptosis through the MAPK pathway. We pretreated BV-2 cells with THC and MAPK inhibitors, including SB203580, U0126, and SP600125 for 1.5 h and then treated the BV-2 cells with 0.1 µg/mL P.a. LPS for 24 h. The cells were stained with annexin V/PI to check for pyroptosis using a flow cytometer. In addition, the culture medium was collected to measure the amount of IL-18 released outside the cells using an ELISA test.

**Figure 5:**
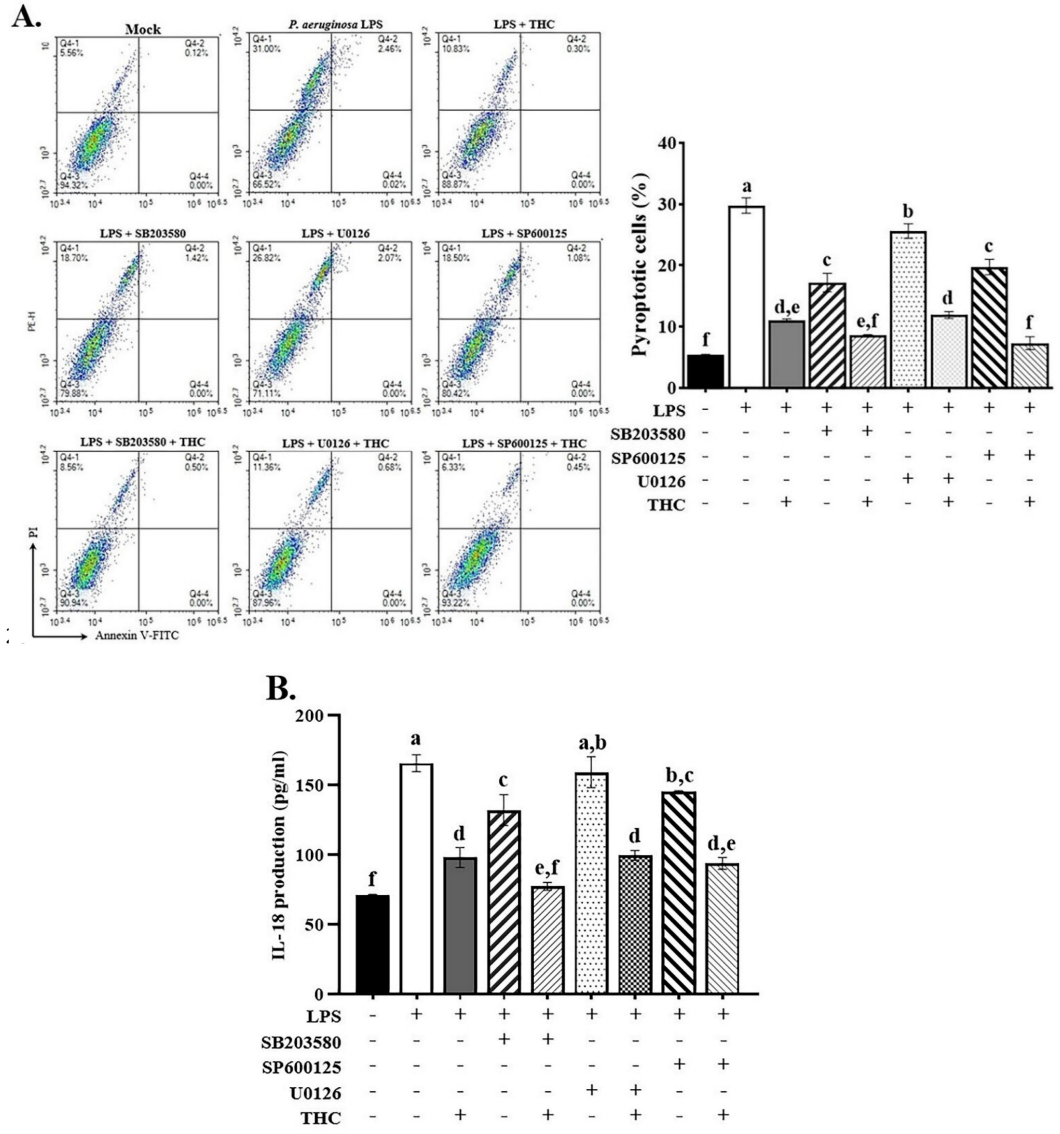
Effects of THC on P.a. LPS-induced pyroptosis in BV-2 cells by MAPK inhibitors. After pretreatment with THC (0 or 40 µM) and SB 203580 (0 or 10 µM), U0126 (0 or 10 µM), or SP 600125 (0 or 20 µM) for 1.5 h and treatment with 0.1 µg/mL P.a. LPS for 24 h,** (A)** BV-2 cells were stained with annexin V and propidium iodide (PI) to isolate pyroptotic cells, and **(B)** extracellular IL-18 levels were measured in the supernatant using ELISA kits. a-f with the same symbol means no statistical difference was observed between groups (p < 0.05).

Treatment with THC or MAPK inhibitors markedly reduced the number of pyroptotic cells, with ERK inhibitors showing the least pronounced effect. The combined administration of THC and these inhibitors led to an even greater suppression of P.a. LPS-induced pyroptosis (Fig. 5A). The ELISA data demonstrated that p38 and JNK inhibitors significantly reduced extracellular IL-18 levels, whereas the ERK inhibitor did not produce a significant change. Likewise, co-treatment with THC and the inhibitors led to a further reduction in IL-18 levels (Fig. 5B). These data indicate that THC reduces p38 and JNK expression in LPS-treated BV-2 cells, implying that the regulation of NLRP3 inflammasome-mediated pyroptosis by THC may be related to p38 and JNK MAPK-dependent inflammatory signaling.

### 3.6. THC alleviated ROS-mediated pyroptosis in P. aeruginosa LPS-induced BV2 cells

**Figure 6:**
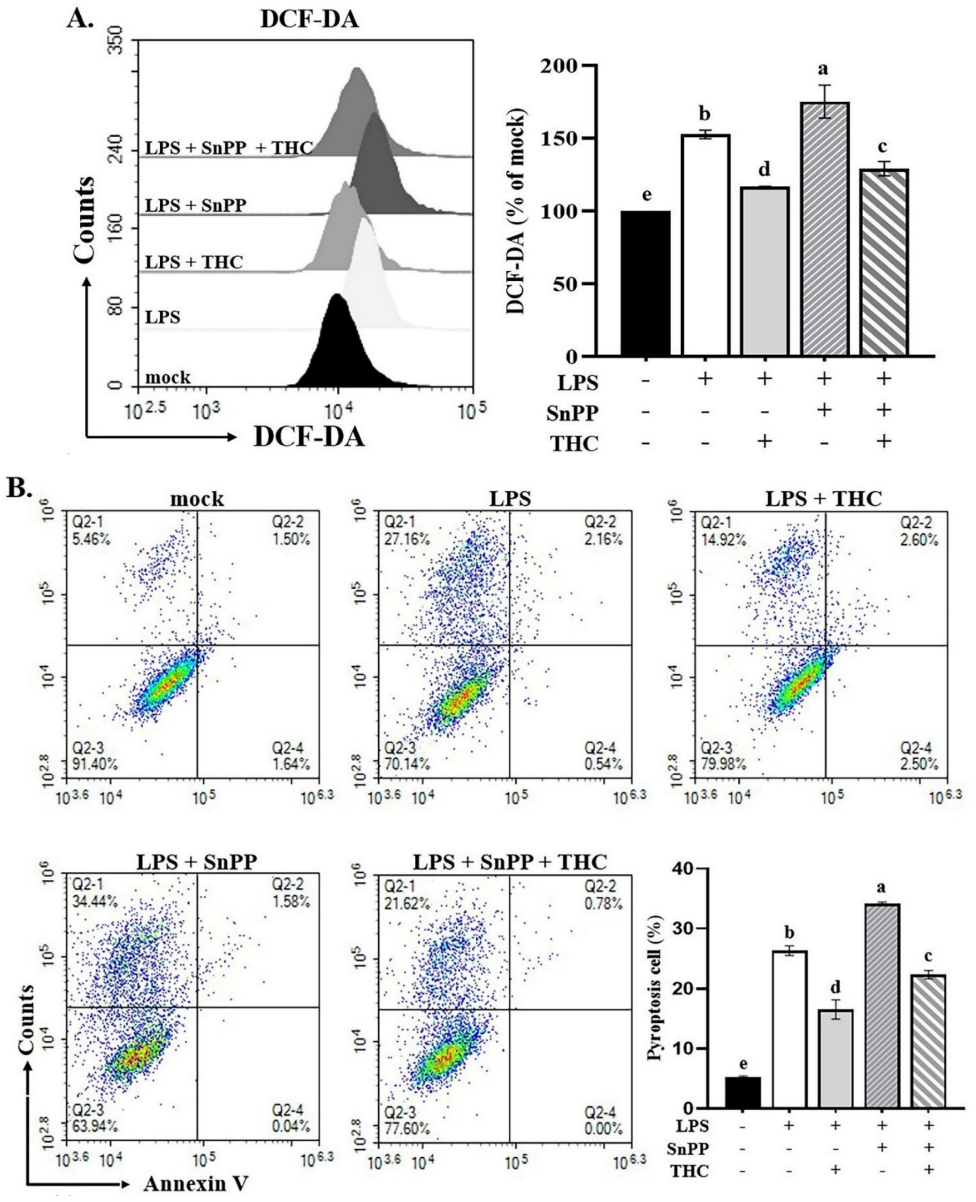
Effects of THC on ROS production and pyroptosis in BV-2 cells with or without HO-1 inhibitor. After pretreatment with THC (0 or 40 µM) and with HO-1 inhibitor SnPP (0 or 20 µM) for 1.5 h and treatment with 0.1 µg/mL P.a. LPS for 24 h, BV-2 cells in each group were **(A)** stained with H_2_DCF-DA to measure intracellular ROS levels or** (B)** stained with annexin V and propidium iodide (PI) to isolate pyroptotic cells. DHICA fluorescence intensity was quantified as a percentage compared with the control group (mock). a-e with the same symbol means no statistical difference was observed between groups (p < 0.05).

In our previous studies, we have shown that THC moderates LPS-induced inflammatory responses through the Nrf-2/HO-1 signaling pathway 15. Park et al. demonstrated that the Nrf-2/HO-1 signaling pathway was involved in decreasing LPS-induced ROS production in BV-2 cells 30. It is not clear whether THC can protect BV-2 cells from ROS-mediated inflammasome activation induced by P.a. LPS through the Nrf-2/HO-1 signaling pathway. We further examined the effects of THC and the HO-1 inhibitor SnPP (20 μM) 15 on ROS production and pyroptosis. As shown in Fig. 6A, pretreatment with THC significantly reduced ROS levels in P.a. LPS-LPS-stimulated BV-2 cells, whereas SnPP increased ROS levels. Interestingly, co-treatment with THC and SnPP attenuated this ROS elevation. Consistently, Fig. 6B demonstrates that SnPP markedly enhanced pyroptosis, while co-administration of THC suppressed this effect. These findings indicate that THC inhibits ROS-mediated NLRP3 inflammasome activation through the p38/JNK and Nrf-2/HO-1 signaling pathways.

## 4. Discussion

As the global population ages, the prevalence of neurodegenerative diseases is expected to continue increasing. Neurodegeneration refers to the complex process of progressive degeneration or abnormal death of neurons, resulting in a series of incurable and debilitating diseases 31. From a pathological perspective, neuronal loss associated with gliomas, protein misfolding and deposition, resulting in abnormal filamentous deposits is the main symptom of NDs 32. There has been evidence that neuronal degeneration is related to pyroptosis, which is mediated by the NLRP3 inflammasome 33. Excessive pyroptosis can lead to tissue damage and plays a crucial role in infectious illnesses 20. LPS-treated BV-2 cells have been used to mimic brain microglial inflammation patterns for many years 34. Curcumin has been shown to protect against apoptosis and to have antioxidant and anti-inflammatory effects in* in vitro* models of various diseases; however, curcumin is not highly bioavailable *in vivo* 35, 36. Aggarwal et al. and Zhang et al. have revealed that THC, a natural derivative of curcumin, retains the biological activities of curcumin and has the advantages of permeability and residence time *in vivo* 11, 12. We previously demonstrated the protective effect of THC on P.a. LPS-induced inflammation by inhibiting the JAK/STAT and Nrf2/HO-1 pathway in microglia 15. However, whether THC can inhibit P.a. LPS-induced NLRP3 inflammasome leading to pyroptosis in microglia remains unclear, so we designed this study to explore these effects.

Pyroptosis is an inflammatory form of cell death characterized by the activation of the NLRP3 inflammasome, which transforms procaspase-1 into cleaved caspase-1 and promotes the GSDMD to generate GSDMD N-termini (GSDMD-N) then the release of proinflammatory cytokines such as IL-1β and IL-18 37. In this study, we found that P.a. LPS induced pyroptosis in BV-2 cells, while THC pretreatment significantly suppressed this effect (Fig. 1A). Furthermore, THC also reduced the expression of key NLRP3 inflammasome-related proteins (including NLRP3, ASC, cleaved caspase-1, and GSDMD-N) in BV-2 cells stimulated with P.a. LPS (Fig. 1B). In our study, IL-18 levels were also assessed, and the results confirmed that THC reduced IL-18 concentrations in P.a. LPS-stimulated BV-2 cells, further supporting its inhibitory effect on inflammasome activation. (Figure 2A and 2B). These findings confirm that THC can inhibit LPS-induced NLRP3 inflammasome-mediated pyroptosis.

Cells were stained with acridine orange to study the integrity of lysosomes, which accumulate in acidic compartments and emit red fluorescence. Our results showed that P.a. LPS increased red fluorescence, whereas THC pretreatment reduced this effect (Fig. 2C), suggesting that THC could prevent the formation of lysozyme. Previous studies have shown that nicotine, free fatty acids, and *Lactobacillus casei* wall components can trigger NLRP3 activation through lysosomal damage and the release of cathepsin B 38; 39. In our results, we also found that THC could inhibit the expression of cathepsin B (Fig. 2D). Therefore, we speculated that THC might inhibit NLRP3 activation by reducing the release of cathepsin B through reducing lysozyme production.

ROS are recognized as key triggers of NLRP3 inflammasome activation 40. In this study, we observed that P.a. LPS stimulation increased ROS levels in BV-2 cells, an effect that was reversed by THC pretreatment (Fig. 3A). The H₂O₂ a type of ROS, plays an important role in various cellular functions. Under physiological conditions, it is efficiently broken down by cellular enzymes to prevent oxidative damage. However, in response to cellular injury, H₂O₂ levels can rise sharply, leading to oxidative stress and potential cellular dysfunction 41, 42. The SOD converts superoxide anions (O₂^•-^) into H₂O₂ and oxygen, while catalase and glutathione further break down H₂O₂ into water and oxygen 43. Our results show that P.a. LPS increases ROS production by elevating H₂O₂ levels and inhibiting GSH activity in BV-2 cells. THC pretreatment counteracts this effect by enhancing GSH activity and reducing H₂O₂ accumulation (Fig. 3B-D). These findings suggest that THC reduces ROS production by inhibiting SOD and enhancing GSH, thereby suppressing NLRP3 inflammasome activation and ultimately preventing pyroptosis.

Excessive ROS production activates MAPK pathways and triggers inflammatory signaling in microglia 44. Studies have shown that ROS-dependent MAPK signaling plays a key role in regulating NFκB transcription and inflammasome formation in LPS-stimulated BV-2 cells 45, 46. In our study, THC promoted the phosphorylation of p38 and JNK, but had minimal effect on ERK in BV-2 cells stimulated with *P. a.* LPS (Fig. 4). As shown in Fig. 5A, treatment with the p38 inhibitor SB203580 and the JNK inhibitor SP600125 significantly reduced IL-18 levels, whereas the ERK inhibitor U0126 did not show a notable effect (Fig. 5B). We also found that all three inhibitors reduced pyroptosis, but U0126 was the least effective (Fig. 5A). These findings suggest that THC regulates NLRP3 inflammasome-mediated pyroptosis primarily through p38- and JNK-dependent MAPK signaling in *P. a.* LPS-stimulated BV-2 cells.

ROS can activate the NLRP3 inflammasome and aggravate the subsequent inflammatory cascade; however, this phenomenon can be inhibited by activating Nrf-2 47. Nrf-2 modulates inflammation by upregulating HO-1, which has anti-inflammatory effects, and inhibiting NLRP3 inflammasome activation through ROS reduction. Previous studies have shown that THC can upregulate the Nrf-2/HO-1 signaling pathway in P.a. LPS-treated BV-2 cells to counteract the production of inflammatory factors IL-6, TNF-α, MIP-2, and IP-10 15; however, whether THC can inhibit NLRP3 inflammation and pyroptosis by activating HO-1 has not been demonstrated. Chen et al. suggested that the inhibition of Nrf-2/HO-1 accelerates the activation of inflammasomes stimulated by ROS 47. This effect was shown to be achieved through the MAPK-mediated NFκB signaling pathway 48. Therefore, we wished to determine whether THC-regulated Nrf-2/HO-1 confers protection against pyroptosis. BV-2 cells were pretreated with the HO-1 inhibitor SnPP alone or combined with THC before P.a. LPS treatment to assess the impact of HO-1 inhibition on pyroptosis. Our results showed that SnPP inhibited HO-1-induced ROS production and pyroptosis, which were inhibited by pretreatment with THC (Figure 6). This indicates that HO-1 activation plays a role in THC's protection against P.a. LPS-induced NLRP3 inflammasome-mediated pyroptosis in BV-2 cells.

**Figure 7:**
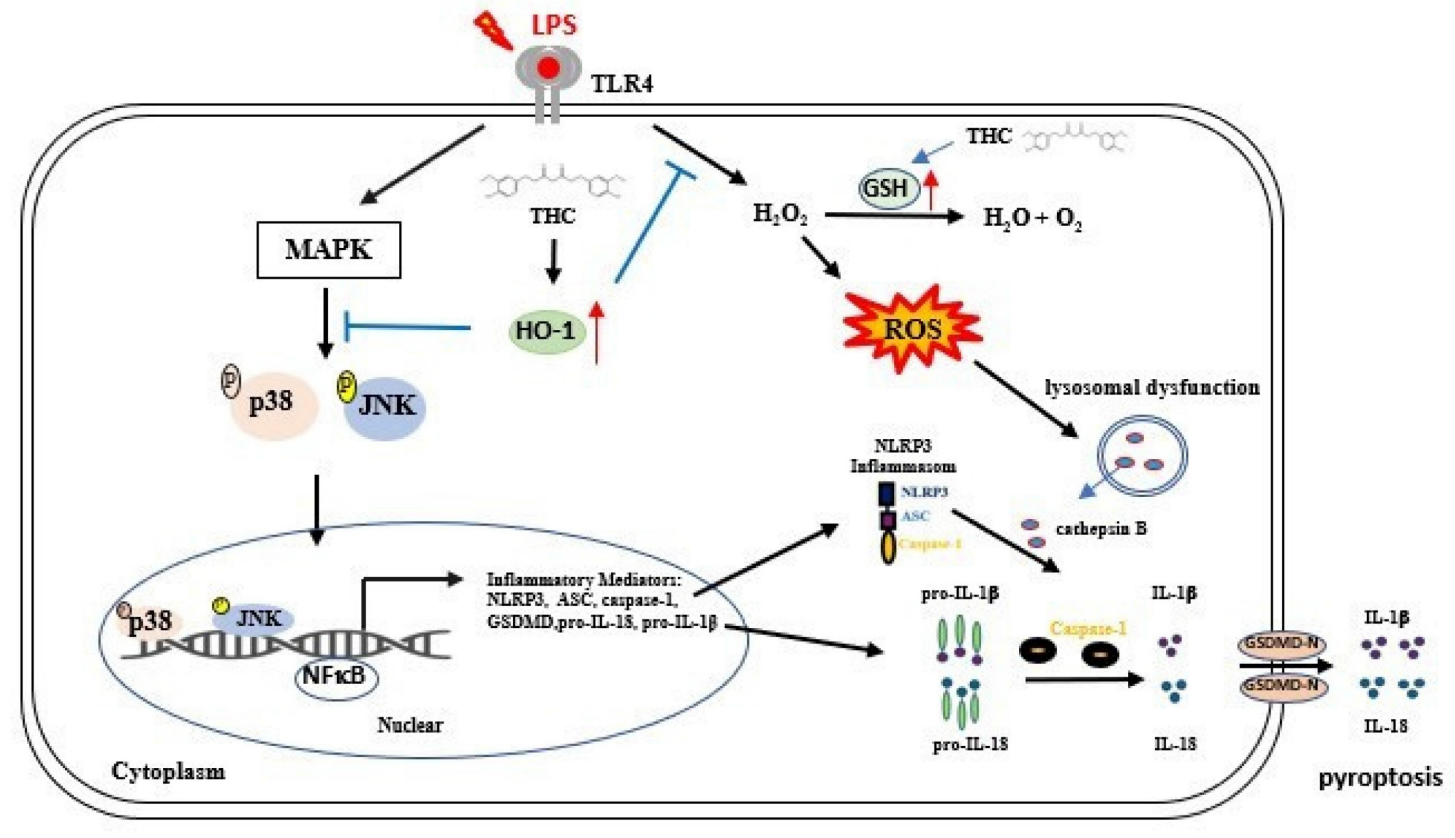
Schematic diagram of THC regulation in P.a. LPS-induced NLRP3 inflammasome led to pyroptosis in BV-2 cells. THC regulates the NLRP3 inflammasome signaling pathway at different levels. P.a. LPS induction NLRP3 inflammasomes-associated proteins through the MAPK/NFκB signaling pathway and inhibits GSH activity, thus raising intracellular H_2_O_2_ levels causing ROS accumulation. Additionally, the induction of lysosomal dysfunction release of cathepsin B triggers the activation of the NLRP3 inflammasome leading to pyroptosis, resulting in the release of IL-1β, and IL-18. However, THC inhibited NLRP3 inflammasome activation leading to pyroptosis by inhibiting the p38/JNK/NFκB signaling pathway and inducting GSH activation alleviated ROS production within cells through the signaling pathway Nrf-2/HO-1, thus inhibiting pyroptosis.

In conclusion, this study provides the first evidence that THC attenuates NLRP3 inflammasome-mediated pyroptosis in P.a. LPS-stimulated BV-2 microglial cells through the inhibition of the p38 and JNK MAPK signaling pathways. Furthermore, THC suppresses ROS-induced pyroptosis by upregulating the expression of antioxidant molecules GSH and HO-1 (Fig. 7). Collectively, these findings highlight the anti-inflammatory properties of THC in microglial cells and suggest its therapeutic potential for the treatment of neuroinflammatory and neurodegenerative disorders.
